# Conversion of Starchy Waste Streams into Polyhydroxyalkanoates Using *Cupriavidus necator* DSM 545

**DOI:** 10.3390/polym12071496

**Published:** 2020-07-04

**Authors:** Silvia Brojanigo, Elettra Parro, Tiziano Cazzorla, Lorenzo Favaro, Marina Basaglia, Sergio Casella

**Affiliations:** Department of Agronomy, Food, Natural Resources, Animals and Environment (DAFNAE), University of Padova, Agripolis, 35020 Legnaro (PD), Italy; silvia.brojanigo@phd.unipd.it (S.B.); elettra.parro@studenti.unipd.it (E.P.); tiziano.cazzorla@gmail.com (T.C.); marina.basaglia@unipd.it (M.B.); sergio.casella@unipd.it (S.C.)

**Keywords:** polyhydroxyalkanoates, optimized saccharification, starchy waste, simultaneous saccharification and fermentation, low-cost carbon source, 3HB

## Abstract

Due to oil shortage and environmental problems, synthetic plastics have to be replaced by different biodegradable materials. A promising alternative could be polyhydroxyalkanoates (PHAs), and the low-cost abundant agricultural starchy by-products could be usefully converted into PHAs by properly selected and/or developed microbes. Among the widely available starchy waste streams, a variety of residues have been explored as substrates, such as broken, discolored, unripe rice and white or purple sweet potato waste. *Cupriavidus necator* DSM 545, a well-known producer of PHAs, was adopted in a simultaneous saccharification and fermentation (SSF) process through an optimized dosage of the commercial amylases cocktail STARGEN™ 002. Broken rice was found to be the most promising carbon source with PHAs levels of up to 5.18 g/L. This research demonstrates that rice and sweet potato waste are low-cost feedstocks for PHAs production, paving the way for the processing of other starchy materials into bioplastics.

## 1. Introduction

Environmental problems regarding plastic pollution have increased in the last few years [1]. In Europe alone, which is the second-largest producer of fossil plastic in the world after China, its production reached 61.8 million tons in 2018 [2]. The bulk of plastic pollution is concentrated in the oceans [3], as demonstrated by the Great Pacific Garbage Patch in the Pacific Ocean. For these reasons, in order to overcome economic and environmental problems linked to plastic pollution, the research focused on the production of innovative bio-based materials such as polyhydroxyalkanoates (PHAs) [4,5,6]. PHAs are a family of eco-friendly polyesters which differ in their properties on the basis of their chemical composition [7,8,9]. Since they are thermoplastic, completely biodegradable and eco-compatible, they could be potential candidates for applications not only in the medical field [10], but also in packaging materials [11,12] and agriculture [13]. These polymers could be accumulated by different bacterial species as carbon energy storage [14,15,16]. Indeed, the accumulation of PHAs occur when strains grow in an unbalanced environmental condition, such as limitation of macroelements and a rich carbon source [17]. The price of PHAs is between 2.4 and 5.5 USD/kg, while the cost of petroleum-based plastics ranges from 0.8 and 1.2 USD/kg [18]. Thus, PHAs substitution of conventional plastics is limited by their expensive manufacturing; in fact, the substrates alone represent up to 50% of the total production costs [13]. Traditional methods for PHAs production involve the use of expensive carbon sources such as pure sugars; therefore, making PHAs more competitive [19,20] and searching for suitable and cheap feedstocks together with new operating strategies [21,22] are the main priorities to be addressed.

Great volumes of organic waste streams coming from agroindustry sectors are yearly available [21,23,24] and could be used as a low-cost feedstocks for the production of high-value bio-products [25,26,27,28,29,30,31]. Recent studies indicate that it is possible to reduce the cost of PHAs manufacturing by using cheap substrates, such as waste streams from agriculture or the food industry [5,21,24,32]. Every year, millions of ton of rice by-products are wasted, with relevant environmental concerns [33]. According to FAOSTAT (Food and Agriculture Organization Corporate Statistical Database), about 782 million tons of rice were globally produced in 2018. Rice milling by-products could be sorted into two groups: starch-(i.e., broken, unripe and discolored rice) and lignocellulosic-rich (i.e., rice husk and rice straw) substrates [34]. Since lignocellulosic materials are the main waste of rice processing, many works are available in the literature [35,36,37,38,39,40]. However, these materials require expensive and complex pre-treatments to saccharify cellulose and hemicellulose into simple sugars before their conversion into PHAs. Focusing on starch-rich rice by-products, the worldwide availability is about 45, 30, and 7 million tons of broken, unripe and discolored rice, respectively [39]. These materials could be abundantly available as carbon sources for PHAs production with simpler pre-treatments. A small number of works report starch-rich substrates, mainly broken rice. Ugwu et al. used broken rice to feed *C. necator* JCM 11282 in a SSF (simultaneous saccharification and fermentation) process, but the substrate was previously liquefied with α-amylase and CaCl_2_ at 50 °C for 1 h, and a glucoamylase was then added [41]. Therefore, all these pre-treatment steps clearly increased the final PHAs production costs.

Another great amount of starchy feedstock is obtained from sweet potato processing. According to FAOSTAT, 92 million tons of sweet potatoes were produced in 2018. After industrial sweet potato processing, 23% of the total product becomes waste. Few studies have reported the conversion of potato waste or peels into PHAs, whereas no research paper has focused yet on the pulp of sweet potato residues. For instance, Haas and colleagues applied *Ralstonia eutropha* NCIMB 11599 in a fed-batch reactor with saccharified potato waste resulting in high 3-hydroxybutyrate (3HB) yields [42]. Gowda and Shivakumar treated potato waste in a separated hydrolysis and fermentation (SHF) set up using *Bacillus* sp. as a PHAs producer [43].

*C. necator* DSM 545 is a well-known PHA producer, but it lacks in amylolytic activities [20,44,45].

As such, starch has to be firstly hydrolyzed into glucose. Broken, discolored, unripe rice and the pulp of white and purple sweet potato by-products were then used as feedstocks in an SSF process. In the SSF process, the enzymatic hydrolysis of the starchy substrates is combined with the fermentation of the released glucose. Since the entire process occurs in a single reactor, the final costs are much lower than those of SHF, which requires more complex equipment and higher energy inputs [46]. The commercial enzymatic cocktail STARGEN™ 002 was supplemented to hydrolyze starch into glucose, which can be used by *C. necator* DSM 545 as a carbon source. PHAs production by *C. necator* DSM 545 in the presence of an equivalent amount of glucose was also considered.

As such, the aim of this work is to evaluate starch-rich waste as a potential feedstock for the growth and PHAs production by C. necator DSM 545 with a low cost process. This is the first study to report the SSF conversion of discolored, unripe rice and white and purple sweet potato waste substrates into PHAs by *C. necator* DSM 545. Moreover, this research paper is a great platform towards the one-step processing of starchy substrates into PHAs by means of an engineered *C. necator* DSM 545 strain for the co-expression of α-amylase and/or glucoamylase, which is currently under development in our laboratories.

## 2. Materials and Methods

### 2.1. Bacterial Strain and Starchy Substrates

*C. necator* DSM 545 provided by DSMZ (Deutsche Sammlung von Mikroorganismen und Zellkulturen, Germany) was used in this work.

Broken, discolored and unripe rice waste was obtained from La Pila (Isola della Scala, Italy). Rice by-products were dried in a fan oven at 60 °C for 48 h, cooled at room temperature for 24 h, and ground in a hammer mill with a 1.00 mm screen.

White and purple sweet potato pulp wastes were obtained from “Lucio Toniolo” Padova University’s agricultural farm. Sweet potato pulp was frozen at −20 °C and lyophilized (EDWARDS Lifesciences Italia S.P.A. lyophilizer).

Dry matter content was obtained, after 48 h, by drying the samples in triplicate in an oven at 100 °C. Starchy by-products were then analyzed according to the AOAC (Association of Official Analytical Chemists, 2000) for the content in cellulose, hemicellulose, starch and lignin. Ash content was quantified by the incineration of samples at 550 °C for 4 h. Protein content was valued using the Kjeldahl method by determining the total nitrogen, then multiplying by the 6.25 factor obtained by the nitrogen content in 100 g of proteins.

### 2.2. Enzymatic Loading Optimization

In order to optimize the enzymes dosage for starchy by-product saccharification, all the substrates were treated with STARGEN™ 002 (Genencor, DuPont-Danisco group, Itasca, IL, USA) at three different loading levels (low, medium and high) according to the instructions of the enzyme supplier. STARGEN™ 002 has an enzymatic activity of 570 GAU/g (GAU, glucoamylase unit) and it is composed of *Aspergillus kawachii* α-amylase expressed in *Thricoderma reesei* and *T. reesei* glucoamylase with a specific gravity of 1.14 g/mL.

The enzymatic saccharification was performed by adding 3% of broken, discolored, unripe rice and white, purple sweet potato in a 250 mL flask which was shaken gently (100 rpm) at 30 °C for 96 h after adjusting the pH to 4 with HCl (1 M). Three dosages of STARGEN™ 002, (1.06, 1.42, and 1.77 μL per gram of starch, hereafter referred to as low, medium, and high enzyme loading) were evaluated. Samples were withdrawn after 0, 12, 24, 36, 48, 60, 72, and 96 h, heat-denatured (boiled for 10 min) to inactivate the enzymes, then kept at −20 °C until HPLC (high performance liquid chromatography) analysis.

To calculate the degree of saccharification (DS) of starch, the concentration of glucose available before saccharification was considered.

$$DS_{starch}=\frac{\left[glucose\;g/L\right]\times 0.9\;}{\left[starch\;g/L\right]}\times 100\%$$

*DS_starch_* was calculated considering the concentration of the total sugar released during hydrolysis when compared to the initial content of starch in the substrates. Since during hydrolysis a water molecule was added, a conversion factor of 0.9 (162/180) was applied due to the difference in the mass between glucose and the anhydroglucose ring [47].

### 2.3. Scanning Electron Microscopy (SEM) Analysis

SEM images were obtained of broken rice samples subjected to SSF by STARGEN™ 002 and *C. necator* DSM 545. Sample preparations were resuspended in 95% ethanol and applied to a specimen stub. Samples were then coated with gold and observed using a Jeol JSM-6490 Scanning Electron Microscope at 15 kV [47].

### 2.4. Culture Media and Production of PHAs from Simultaneous Saccharification and Fermentation (SSF)

All media and starchy substrates were autoclaved at 121 °C for 20 min.

Cells of *C. necator* DSM 545 were maintained on nutrient agar (g/L: peptone 15, yeast extract 3, NaCl 6, glucose 1, agar 15).

Pre-inoculum of *C. necator* DSM 545 was obtained in DSMZ81 medium (DSMZ, Germany) with 30 g/L glucose in aerobically conditions, at 30 °C, under shaking (145 rpm). DSMZ81 medium contains NH_4_Cl 1 g/L, MgSO_4_ 7H_2_O 0.5 g/L, NaHCO_3_ 0.5 g/L, KH_2_PO_4_ 2.3 g/L, Na_2_HPO_4_ 7H_2_O 2.9 g/L, CaCl_2_ 2H_2_O 0.01 g/L, ferric ammonium citrate 0.05 g/L and standard vitamin solution, which contains the following: riboflavin 0.0005 mg; thiamine-HCl·2H_2_O 0.0025 mg; nicotinic acid 0.0025 mg; pyridoxine-HCl 0.0025 mg; Ca-pantothenate 0.0025 mg; biotin 0.000005 mg; folic acid 0.00001 mg; vitamin B_12_ 0.00005 mg. After 24 h, cells were harvested by centrifugation (5500 rpm for 15 min) and washed twice with NaCl 0.9% to remove any carbon sources.

In the case of SSF experiments, cells were aerobically inoculated (initial OD_600 nm_ = 0.3) at 30 °C under shaking (145 rpm) in a 250 mL flask containing 100 mL DSMZ81 medium and 3% of broken, unripe, discolored rice and white and purple sweet potato individually, as the only carbon source. An optimized dosage of STARGEN™ 002 was added to promote starch hydrolysis and experiments without STARGEN™ 002 were also included for each sample. As a benchmark, an experiment with 3% glucose as a carbon source was evaluated. After 72 and 96 h, cells were centrifuged (5500 rpm for 15 min) and pellets were stored at −80 °C before being lyophilized for PHAs analysis.

Broken rice, selected as one of the most promising substrates, was adopted also in SSF for 168 h and an experiment with glucose, as a benchmark, was also assessed. In this case, PHAs production was evaluated every 24 h.

All experiments were performed in triplicate and the standard deviation is reported.

### 2.5. PHAs Analysis

PHAs concentration was determined according to Torri et al. [48] and, mainly, Braunegg et al. [49].

Samples of lyophilized cells (10 mL) were treated at 100 °C for 4 h. The propyl esters of hydroxylalkanoic acids were analyzed by gas chromatography using a Thermo Finnigan Trace GC, equipped with a flame ionization detector (FID) and AT-WAX column (30 m × 0.25 mm × 0.25 μm). The gas carrier was helium at a flow rate 1.2 mL/min and the split/splitless injector with a split ratio of 1:30 was set at 250 °C. The FID and oven temperature was set at 270 and 150 °C, respectively. Benzoic acid was used as an internal standard whereas the external standards, 3-hydroxybutyric acid (3HB), poly(3-hydroxybutyric acid-co-3-hydroxyvaleric acid P(3HB-co-12 mol% 3HV) and poly(3-hydroxybutyric acid-co-4-hydroxybytyric acid) P(3HB-co-11.2 mol% 4HB), were purchased from Sigma-Aldrich (Italy) [50].

The results were expressed as the percentage of PHAs on cell dry biomass (CDM) or grams of PHAs/liter of culture. Selected chromatograms are available in the Supplementary Figures S1–S3.

## 3. Results and Discussion

### 3.1. Starchy Waste Composition

Rice by-products and sweet potato waste composition are reported in Table 1.

For the rice substrates tested, starch was abundant in discolored and broken rice, whereas slightly lower amounts were found in unripe rice (68.58%). Protein is the second prominent fraction, while the content of ash, cellulose and hemicellulose are not relevant.

The starch content for sweet potato substrates was lower than that detected for rice by-products. In this case, the chemical composition reveals an amount of ash and cellulose higher than that of rice substrates, but less than 8% of protein. Lignin content was less than 1%.

Overall, the compositions of the tested starchy substrates are in agreement with those reported in the literature, and clearly confirmed that starch is the main polysaccharide in the selected substrates. Nunes et al. and Favaro et al. reported a starch content for broken rice of around 77%, with proteins between 7.5–8.5% [39,51]. Yokoi et al., analyzing the chemical composition of sweet potato waste, confirm a starch content of up to 50% [52].

With the final objective to convert directly rice by-products and sweet potato waste into PHAs, *C. necator* DSM 545, a well-known and efficient PHAs producer, was adopted to process these waste streams into PHAs. The strain exhibited very limited biomass growth (ranging from 0.5 to 0.9 g/L) and PHAs production with values of up to 0.02 g/L. This finding further confirmed that *C. necator* DSM 545 lacks amylolytic enzymes [20]; thus, the SSF strategy was selected to process each starchy by-product into PHAs.

### 3.2. Optimization of Enzymatic Saccharification of Starchy Substrates

**Figure 1:**
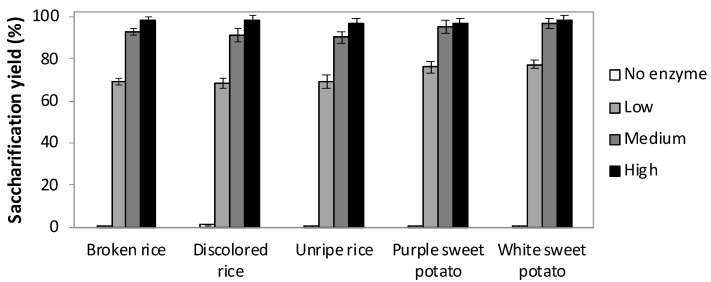
Saccharification yields at 96 h of 3% (*w*/*v*) starchy substrates with three different loadings of STARGEN™ 002: 1.06 (low), 1.42 (medium) and 1.77 (high) µL per g of starch. Values represent the mean of three replicates and error bars represent the standard deviation.

To optimize the enzymatic dose for SSF experiments, each starchy by-product was hydrolyzed by three different loadings of STARGEN™ 002: 1.06 (low), 1.42 (medium) and 1.77 (high) µL per g of starch. As expected, the only pre-treatment in autoclave resulted in low total sugar release. When combined with enzymatic hydrolysis, the higher enzyme dose, the greater obtained saccharification yields (Figure 1).

**Figure 2:**
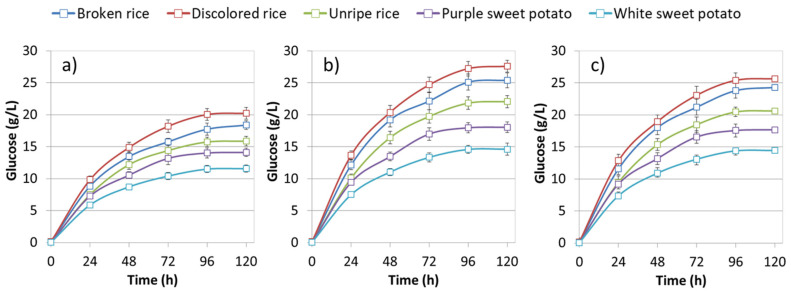
Glucose release (g/L) under saccharification of different starchy substrates (3% *w*/*v*) with increasing loadings of STARGEN™ 002: (**a**) 1.06 (low), (**b**) 1.42 (medium) and (**c**) 1.77 (high) µL per g of starch.

Most of the glucose, about 75%, was obtained within 48 h in all the three enzymatic loadings, with glucose levels slowly increasing up to 96 h, after which, as indicated in Figure 2, no further enzymatic hydrolysis took place. As expected, the highest glucose releases were detected with rice by-products where starch content was higher than those of sweet potato residues (Table 1).

Within the rice by-products, slightly higher saccharification yields were obtained with the highest STARGEN™ 002 loading, whereas sweet potato residues have been efficiently saccharified by supplementing the medium dosages of 1.42 µL per g of starch (Figure 1). A further increase in commercial enzymes did not result in a significantly higher glucose release (Figure 1 and Figure 2). Since the enzyme represent a relevant cost in the entire process, the medium STARGEN™ 002 loading was selected as the most efficient enzymatic dosage for SSF experiments.

### 3.3. PHA Production by C. necator DSM 545 on Starch-Rich Waste

Since one of the main objectives of the PHAs industry is to reduce the production costs, this study adopted the one-step fermentation process, known to require simpler equipment and procedures. For SSF experiments, *C. necator* DSM 545 was incubated in DSMZ81 broth with 3% starch-rich material or, as a comparison, glucose. Cultures were incubated under shaking at 30°C and 3HB content was quantified after 72 and 96 h of incubation (Table 2).

From rice by-products, *C. necator* DSM 545 accumulated 3HB up to 39.51 and 44.09% of cell dry weight after 72 and 96 h of incubation, respectively. The comparison between 3HB levels from two times of incubation showed a higher yield at 96 h for broken and discolored rice. Instead, for unripe rice, less accumulation occurred when increasing the incubation time. This can be due to the individual chemical compositions of the different rice substrates. Indeed, unripe rice has less starch and more proteins, ashes, cellulose and hemicellulose. This result could also be due to different starch digestibility levels within the tested substrates [53]. For instance, other researchers have already described that unripe rice could resist enzymatic hydrolysis longer than well-polished rice [39,54].

The others starchy substrates processed to PHAs in this work were white and purple sweet potato waste, showing a similar chemical composition (Table 1). In this experiment, the additional incubation time to 96 h did not result in higher 3HB accumulation (Table 2). Purple sweet potato supported higher PHAs levels with 34.42% of cell dry weight. This can be due to its higher starch content as compared to that of white sweet potato (Table 1). Other studies carried out with potato waste in bioreactors report data from 55% [42] to 77% [55] of cell dry weight, but in these experiments the hydrolysis of the potato starch to glucose was obtained before the strain inoculation. Moreover, these data are referred to as potato waste, while for sweet potato residues, data are not available in the literature for comparison.

Since glucose is the preferred substrate for the growth of PHAs and its accumulation by *C. necator* DSM 545 [56], the strain was also grown in DSMZ81 with 3% glucose as a carbon source, thus producing 3HB up to 70 and 77% of cell dry weight with a final concentration of 4.95 and 6.60 g/L after 72 and 96 h, respectively (Table 2).

Overall, comparing all the PHAs performances obtained from rice by-products and sweet potato waste, broken rice seems to be the most promising substrate with 13.32 g/L of cell biomass and 5.18 g/L of 3HB after 96 h.

**Figure 3:**
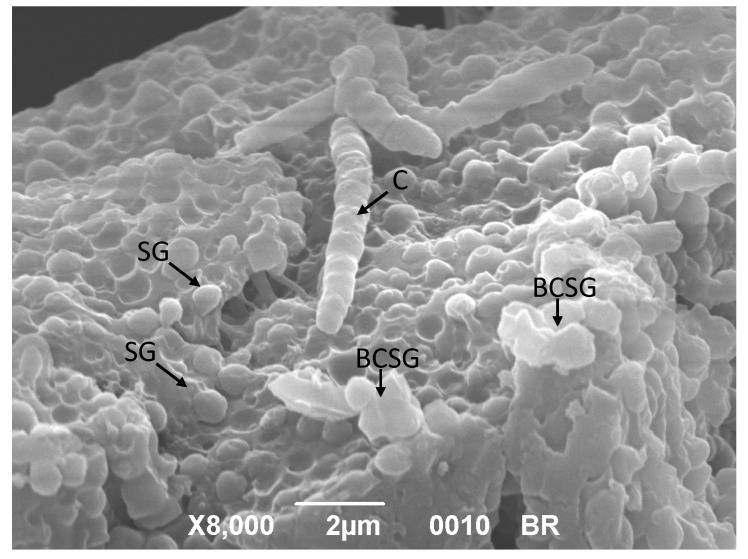
SEM micrograph of 3% *w*/*v* broken rice under the SSF regime with STARGEN™ 002 and *C. necator* DSM 545. *C. necator* DSM 545 (C), starch granules (SG). Broken compound starch granules (BCSG), with evident damages at their original polygonal structure, are visible on rice endosperm.

In terms of g/L, the PHAs production by *C. necator* DSM 545 was indeed close to those detected from glucose. SEM reveals that, after 96 h, the majority of starch granules contained in broken rice seems to be hydrolyzed by the commercial amylases. As reported in Figure 3, only few starch granules (SG) were still visible on the rice endosperm [57]. Moreover, a limited number of broken compound starch granules (BCSG) were observable with evident damages at their original polygonal structure [57]. This is in agreement with the high saccharification yields reported for broken rice in Figure 1.

Noteworthy, the PHAs content was even higher than those reported by Ugwu et al., who obtained 38% of cell dry weight only after multiple and more complex pre-treatments [41].

Excluding unripe rice and white sweet potato, the prolongation of the incubation until 96 h during SSF increase the PHAs yield (Table 2). It is conceivable that time has a positive effect on the accumulation of PHAs, allowing the strain to transform the progressively released glucose into 3HB. Other studies confirm the direct link between time and accumulation of PHAs [58], although, prolonging the process too long causes the depletion of the carbon source and the consumption of PHAs by microorganisms for growing [59,60,61].

**Figure 4:**
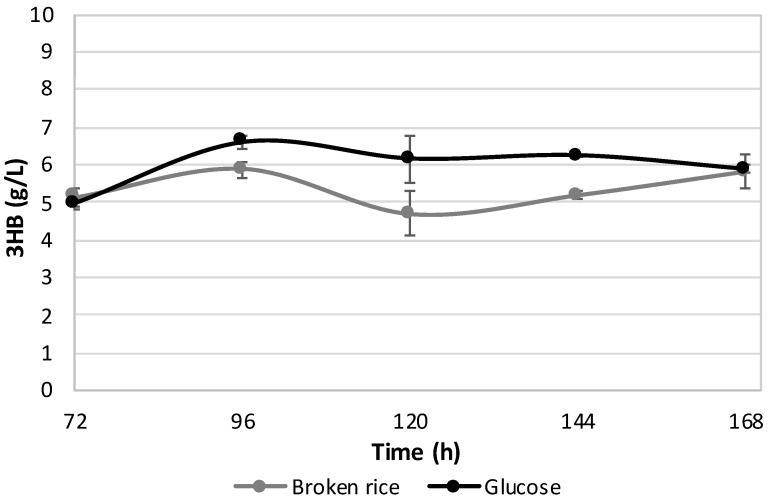
Levels (g/L) of 3HB from 3% broken rice under the SSF regime using the medium dosage of STARGEN™ 002 (1.42 µL per g of starch). An experiment with glucose as a carbon source was included for comparison. The values represent the means of three replicates ± SD.

To further investigate 3HB production from broken rice under the SSF regime, SSF was performed for a longer incubation time (up to 168 h), again in comparison with glucose as a benchmark substrate. However, as reported in Figure 4, this did not result in additional 3HB content, as both the substrates that showed similar 3HB yields. The glucose experiment indicates that the 3HB value at 96 h is not significantly different from those at 120 and 144 h, while in the case of broken rice, this value seems to slightly decrease at 120 h. It will be interesting, at a pre-industrial level, to verify why it happens and to determine if this decrease could be eliminated. For instance, since it is very difficult to control and calibrate the loading of natural heterogeneous substrates, the C/N ratio could fluctuate too much in SSF processes, and the possible action of depolymerases could take place [61].

As such, the incubation time of 96 h was found to be the most suitable to support PHAs production from broken rice and longer incubations, while increasing the total production costs, did not result in additional and significantly higher PHAs contents.

## 4. Conclusions

This study demonstrates that, in a SSF procedure, starchy by-products could be processed as potential carbon sources to grow and produce PHAs by *C. necator* DSM 545. Thus, the substrates were efficiently hydrolyzed by optimized dosages of enzymes and then converted into PHAs. Broken rice was found to be the most promising substrate with a 3HB yield of up to 5.18 g/L. To further improve the techno-economical feasibility of the process, experiments aimed at the optimization of SSF parameters and, above all, the development of an engineered *C. necator* DSM 545 strain, are in progress. As such, the processing of starchy waste streams into PHAs will be a step closer towards the large-scale application.

## Figures and Tables

**Table 1:** Chemical composition of rice and sweet potato waste streams used in this study.

		(% DM)					
Substrate	% DM ^1^	Starch	Protein	Ash	Cellulose	Hemicellulose	Lignin
Broken rice	88.11	77.74	8.31	0.47	0.22	0.54	-
Discolored rice	87.87	84.61	8.02	0.50	0.11	0.90	-
Unripe rice	87.87	68.58	9.86	1.54	1.80	3.68	-
Purple sweet potato	33.88	55.79	7.70	4.51	3.22	1.65	0.79
White sweet potato	29.37	44.66	7.17	3.74	3.31	1.07	0.60

^1^ DM: dry matter.

**Table 2:** Production of 3-hydroxybutyrate (3HB) by *C. necator* DSM 545 under the simultaneous saccharification and fermentation (SSF) experiments with 3% of rice by-products or sweet potato waste and a medium dosage of STARGEN™ 002 (1.42 µL per g of starch). As a benchmark, an experiment with 3% of glucose was also included. The values represent the means of three replicates ± SD.

Substrate	CDM ^1^ (g/L)	Time (h)	3HB (% CDM ^1^)	3HB (g/L)
Glucose	7.08 ± 0.07	72	70.00 ± 0.77	4.95 ± 0.05
	8.51 ± 0.08	96	77.60 ± 0.81	6.60 ± 0.07
Broken rice	12.99 ± 0.06	72	39.51 ± 0.24	5.13 ± 0.03
	13.32 ± 0.09	96	44.09 ± 0.19	5.18 ± 0.02
Discolored rice	8.87 ± 2.17	72	20.10 ± 1.31	1.95 ± 0.13
	11.33 ± 1.32	96	31.69 ± 1.23	3.65 ± 0.14
Unripe rice	11.86 ± 0.24	72	19.39 ± 0.35	2.30 ± 0.04
	11.29 ± 0.16	96	17.81 ± 0.40	2.02 ± 0.05
Purple sweet potato	10.86 ± 1.75	72	31.05 ± 0.73	3.39 ± 0.08
	10.48 ± 0.00	96	34.42 ± 0.17	3.61 ± 0.02
White sweet potato	11.10 ± 0.66	72	27.67 ± 1.11	3.12 ± 0.13
	10.51 ± 0.24	96	22.10 ± 0.87	2.33 ± 0.09

^1^ CDM: cell dry matter.

## Supplementary Materials

The following are available online at https://www.mdpi.com/2073-4360/12/7/1496/s1, Figure S1: Gas chromatography (GC) analysis of 3-hydroxybutyric acid (3HB) methyl ester, after chloroform extraction, in dry cells of *C. necator* DSM 545 grown for 96 h in SSF of broken rice (3% *w*/*v*) and STARGEN™ 002 (1.42 µL per g of starch). GC analysis reveals that 3HB was the only polymer accumulated by *C. necator* DSM 545. I.S. stands for internal standard (benzoic acid). Figure S2: Gas chromatography (GC) analysis of 3-hydroxybutyric acid (3HB) methyl ester, after chloroform extraction, in dry cells of *C. necator* DSM 545 grown for 96 h in SSF of purple sweet potato (3% *w*/*v*) and STARGEN™ 002 (1.42 µL per g of starch). GC analysis reveals that 3HB was the only polymer accumulated by *C. necator* DSM 545. I.S. stands for internal standard (benzoic acid). Figure S3: Gas chromatography (GC) analysis of 3-hydroxybutyric acid (3HB) methyl ester, after chloroform extraction, in dry cells of *C. necator* DSM 545 grown for 96 h in the presence of glucose (3%). GC analysis reveals that 3HB was the only polymer accumulated by *C. necator* DSM 545. I.S. stands for internal standard (benzoic acid).
