# Supplementary figures and images for: Conversion of Starchy Waste Streams into Polyhydroxyalkanoates Using Cupriavidus necator DSM 545
Source: Polymers (Basel). 2020 Jul 4;12(7):1496. doi: 10.3390/polym12071496 (PMC7407217; doi:10.3390/polym12071496)

## Supplementary Figures

Figure S1.

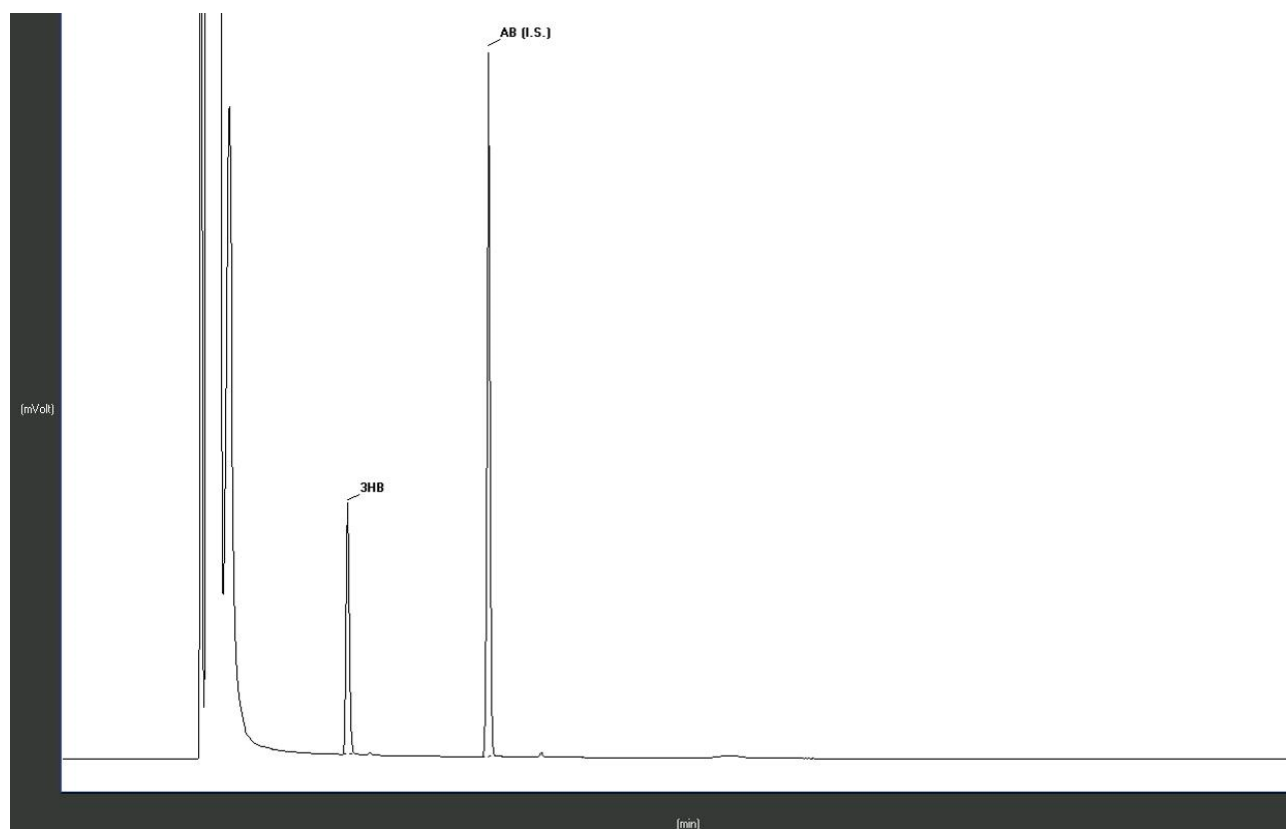

Figure S2.

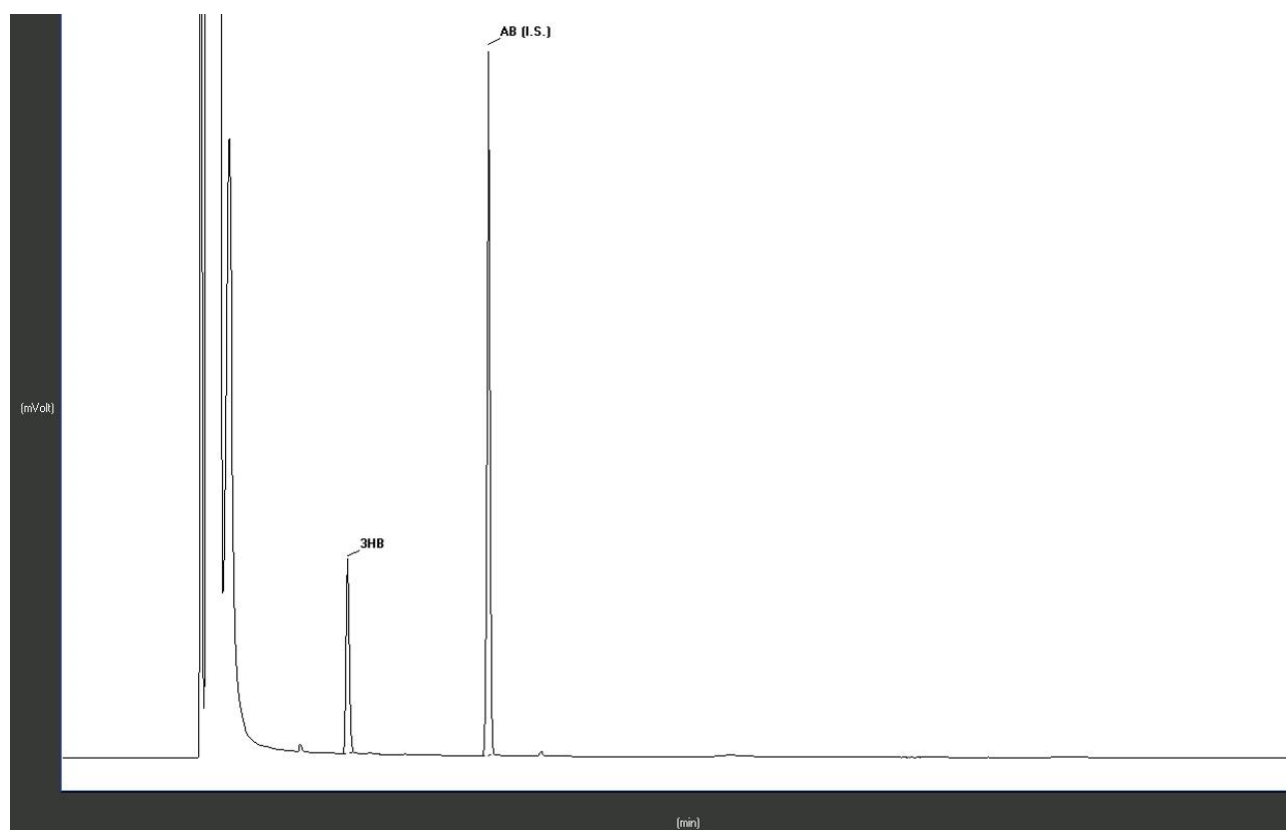

Figure S3.

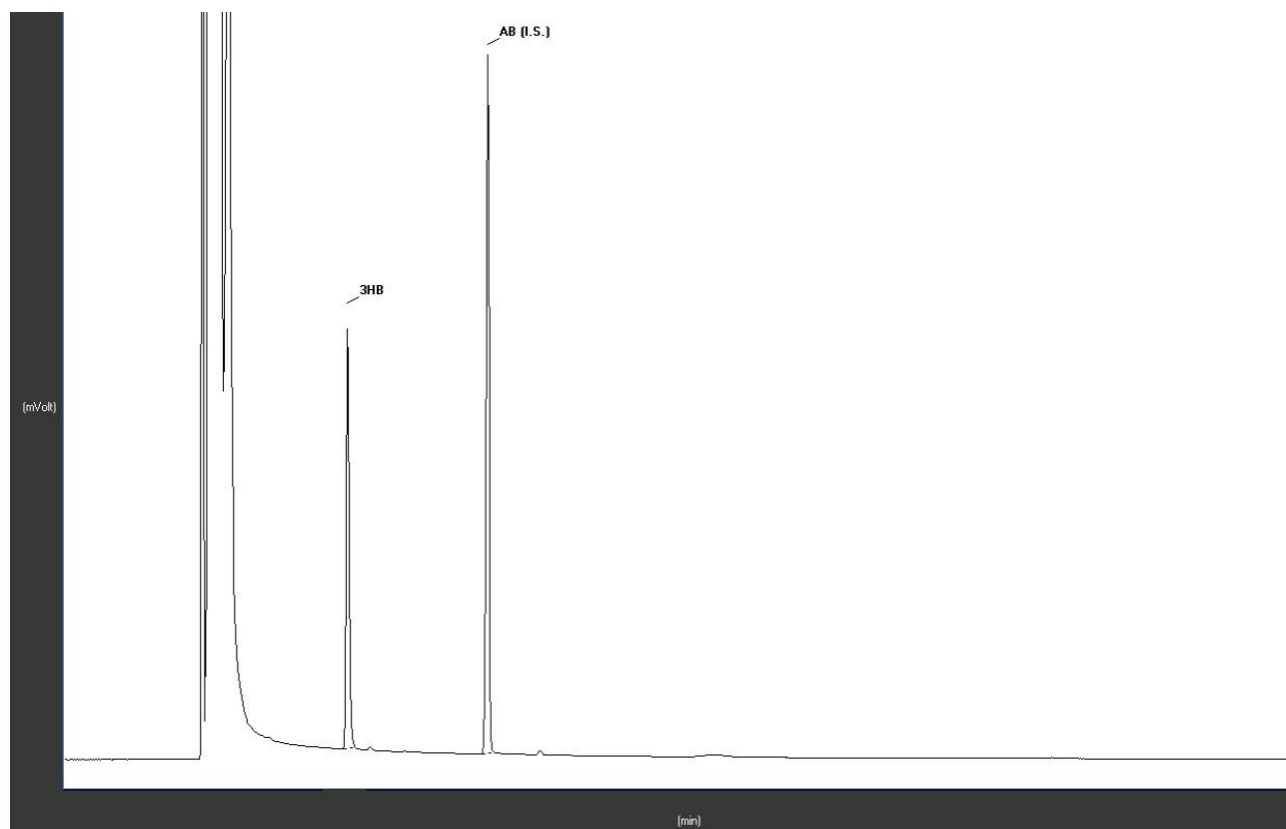

Supplement: Supplementary file 1 [file polymers-12-01496-s001.pdf]
